# Chloroplast (Cp) Transcriptome of *P. davidiana* Dode*×P. bolleana* Lauch provides insight into the Cp drought response and *Populus* Cp phylogeny

**DOI:** 10.1186/s12862-020-01622-7

**Published:** 2020-05-06

**Authors:** Xin Zhang, Chenrui Gu, Tianxu Zhang, Botong Tong, Heng Zhang, Yueliang Wu, Chuanping Yang

**Affiliations:** 1 State Key Laboratory of Tree Genetics and Breeding, Northeast Forestry University, 26 Hexing Road, Harbin, 150040 China; 2 School of Forestry, Shenyang Agricultural University, 120 Dongling Road, Shenyang, 10866 China; 3 College of Life Science, Northeast Forestry University, 26 Hexing Road, Harbin, 150040 China

**Keywords:** Poplar, Chloroplast genome, Cp Transcriptome, Drought stress, Polymorphism

## Abstract

**Background:**

Raw second-generation (2G) lignocellulosic biomass materials have the potential for development into a sustainable and renewable source of energy. Poplar is regarded as a promising 2G material *(P. davidiana Dode×P. bolleana Lauch*, *P. bolleana*, *P. davidiana, P. euphratica*, et al). However, their large-scale commercialization still faces many obstacles. For example, drought prevents sufficient irrigation or rainfall, which can reduce soil moisture and eventually destroy the chloroplast, the plant photosynthetic organelle. Heterosis is widely used in the production of drought-tolerant materials, such as the superior clone “Shanxinyang” selected from the offspring of *Populus davidiana* Dode*×Populus bolleana* Lauch. Because it produces good wood and is easily genetically transformed, “Shanxinyang” has become a promising material for use in tree genetics. It is also one of the most abundant biofuel plants in northern China. Understanding the genetic features of chloroplasts, the cp transcriptome and physiology is crucial to elucidating the chloroplast drought-response model.

**Results:**

In this study, the whole genome of “Shanxinyang” was sequenced. The chloroplast genome was assembled, and chloroplast structure was analysed and compared with that of other popular plants. Chloroplast transcriptome analysis was performed under drought conditions. The total length of the “Shanxinyang” chloroplast genome was 156,190 bp, the GC content was 36.75%, and the genome was composed of four typical areas (LSC, IRa, IRb, and SSC). A total of 114 simple repeats were detected in the chloroplast genome of “Shanxinyang”. In cp transcriptome analysis, we found 161 up-regulated and 157 down-regulated genes under drought, and 9 cpDEGs was randomly selected to conduct reverse transcription (RT)–qPCR., in which the Log2 (fold change) was significantly consistent with the qPCR results. The analysis of chloroplast transcription under drought provided clues for understanding chloroplast function under drought. The phylogenetic position of “Shanxinyang” within *Populus* was analysed by using the chloroplast genome sequences of 23 *Populus* plants, showing that “Shanxinyang” belongs to Sect. *Populus* and is sister to *Populus davidiana.* Further, mVISTA analysis showed that the variation in non-coding (regulatory) regions was greater than that in coding regions, which suggests that further attention should be paid to the chloroplast in order to obtain new evolutionary or functional insights related to aspects of plant biology.

**Conclusions:**

Our findings indicate that complex prokaryotic genome regulation occurs when processing transcripts under drought stress. The results not only offer clues for understanding the chloroplast genome and transcription features in woody plants but also serve as a basis for future molecular studies on poplar species.

## Summary

The *Populus davidiana* Dode*×Populus bolleana* Lauch chloroplast genome, *Populus* chloroplast phylogeny and differentially expressed cpDEGs induced by drought stress examined here offer clues for understanding the regulatory mechanisms of the cp transcriptome.

## Background

Short-rotation period (SRC, SRF) poplar is widely considered to be a promising lignocellulosic material for second-generation biofuel production. It grows rapidly and is widely distributed in the Northern Hemisphere. “Shanxinyang” originated in northern China and is one of the most widely distributed trees in urban areas and forests. The ubiquity of popular varieties symbolizes their ability to adapt to different environmental conditions [1, 2]. Drought causes irrigation or rainfall to be insufficient, thus reducing soil moisture and ultimately harming plants, usually accompanied by a higher transpiration rate through the root than through the plant surface [3]. Aspects of plant physiology, water pressure, water pressure resistance and reclaimed water reuse efficiency in relation to these negative effects are widely researched [4]. Although poplar trees have more and deeper roots than agricultural plants, they are still affected by continuous drought. Different crops respond to drought stress, such as corn [5], potato [6], wheat [7], sugarcane [8] and other woody plants, including poplar, pine and oak [9]. These studies provide useful information for identifying potential mechanisms and managing possible problems, while studies of the chloroplast genome and transcriptome of drought-tolerant species are less common.

It has long been known that multiple cis and trans transcripts are produced by the chloroplast and processed into small mature RNA, which may indicate that chloroplast participation in the behaviour of the organism is limited to only basic functions [10, 11]. However, there are many untranslatable areas in the chloroplast genome (for example, the areas between transcription units, accounting for 40% or more of the total genome), and the operon transcription pattern was found to lead to a stable, fixed-size transcript, showing that chloroplast gene transcription is also regulated. Furthermore, this transcription has been confirmed to participate in many defence reactions and counter-reactions. Previous research has shown that the chloroplast genome expression pattern is similar to the prokaryotic genome expression pattern. However, recent studies have shown that chloroplast genes are mostly functional and regulated [12] and participate in a variety of anti-stress responses [13–16]. A transcription pause phenomenon has been reported in plants, which is regulated by transcription pause factors in chloroplasts [17]. It is of far-reaching significance to explore whole-plastid transcription level changes triggered by drought stress, not only because the whole plastid transcriptome has not been reported in many plants but also because such information would provide a potential mechanistic understanding at the transcriptome level. This information might also provide insight into the functional evolutionary strategy of *Populus,* and phylogenetic analysis may in turn provide a new understanding of why “Shanxinyang” is a drought-tolerant clone.

The relationship between the taxonomy and phylogeny of *Populus* has always been controversial. Scholars generally consider *Populus* to be composed of five factions, which was confirmed by the recent Angiosperm Phylogeny Group IV system [18–20]. In *Populus*, the main basis for the division of the five factions is a morphological classification system and a molecular system based on chloroplast gene fragments. Related scientific research has been carried out in recent years, but it is mostly limited to model species that have been sequenced. In addition, there are many disputes regarding the division of species in this family. Unclear species boundaries directly lead to difficulty in identifying some species in this family [10], and structural analysis of chloroplasts may provide new insights.

DNA barcoding is a technique that uses a standard DNA sequence to identify species quickly. However, for species with close relationships, the resolution of DNA barcodes is relatively low. Recently, research has shown that DNA barcoding cannot completely solve the problem of related species identification [21, 22]. The average length of chloroplast genomes of angiosperms is approximately 150 kb, and the variation is much higher than that in a single DNA barcode sequence, which reveals close relationships. In addition, the evolutionary rates of coding and non-coding regions of the chloroplast genome are quite different, which makes them suitable for the study of different taxonomic levels. With the development of second-generation high-throughput sequencing technology, more chloroplast genomes are being sequenced. However, chloroplast genomic data are still lacking. Currently, there are only 1100 complete chloroplast genomes in the NCBI genome database (https://www.ncbi.nlm.nih.gov/genome/organelle/). More extensive transcriptome data are rarely used in chloroplast research, and analysing the differences between chloroplast coding and non-coding regions is key to understanding chloroplasts.

Only 23 chloroplast genomes have been sequenced in *Populus* and hybrid *Populus*, chloroplast sequences from stress-resistant species are even less reported, and reports of chloroplast transcription under drought conditions are unavailable. In this study, the chloroplast genome of “Shanxinyang” was sequenced, and its structural characteristics and transcriptional behaviour under drought conditions were analysed. Such information will not only offer clues for understanding the chloroplast genome and transcription features in woody plants but also serve as a basis for future molecular studies on poplar species.

## Results

### Genome assembly and structural analysis of the chloroplast

According to the sequencing results obtained with the Illumina platform, a total of 34,780,120 reads were generated. Through comparison with the *Populus* chloroplast genome, approximately 1,887,236 chloroplast genome reads were mapped, the mapping ratio was 5.43, the coverage was 100%, and 3471.03 coverage (depth) was reached. After the chloroplast genome assembly was qualified, it was submitted to the GenBank database under accession number MN190025.

**Fig. 1:**
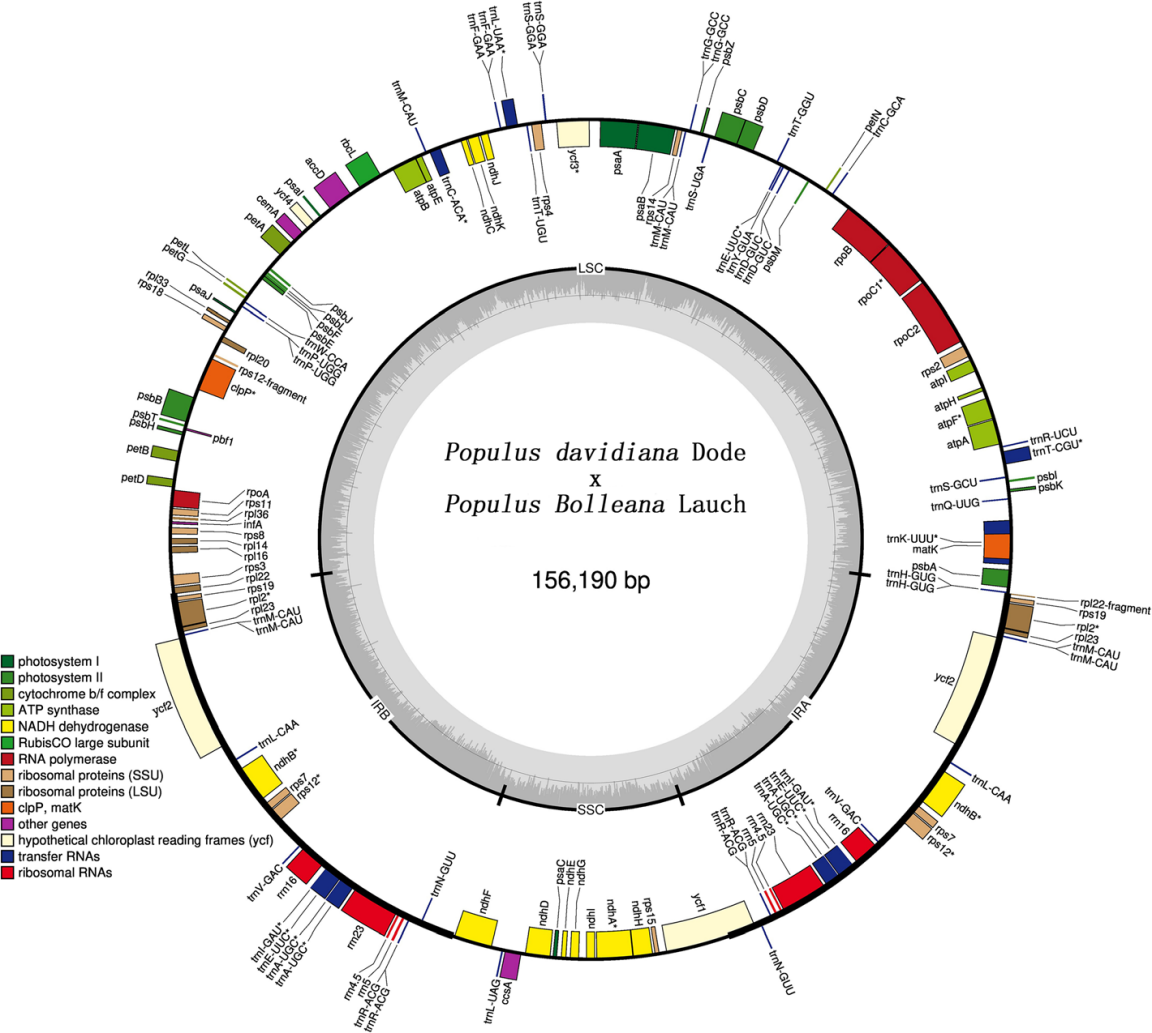
Assembly and annotation of the chloroplast genome of “Shanxinyang”. Genes located outside the outer rim are transcribed in a counterclockwise direction, whereas genes inside the outer rim are transcribed in a clockwise direction. The coloured bars indicate different known functional groups. The dashed grey area in the inner circle shows the GC content percentage of the corresponding genes. LSC, SSC, and IR denote large single copy, small single copy, and inverted repeat, respectively

The total length of the “Shanxinyang” chloroplast genome was 156,190 bp, and the GC content was 36.75%. Its structure was similar to that of most angiosperm genomes, and it was composed of four typical regions: the LSC region, the SSC region and a pair of separated IR regions, with lengths of 84,612, 16,498 and 27,540 bp, respectively (Fig. 1). The GC content was unevenly distributed along the “Shanxinyang” chloroplast genome, with that in the IR region being the highest, which may be responsible for IR region conservation. The total length of the coding region was 78,407 bp, covering approximately 50.20% of the whole chloroplast genome.

There were 131 annotated genes in the “Shanxinyang” chloroplast genome, including 86 protein-coding genes, 37 tRNA genes and 8 rRNA genes, among which 23 tRNA genes, 14 protein-coding genes and 4 rRNA genes were located in the two IR regions (Fig. 1).

Introns play an important role in gene selective splicing. In the “Shanxinyang” chloroplast genome, a total of 16 intron-containing genes, including 8 tRNA genes and 8 protein-coding genes, were inserted and released. Except for the *clpP* and *ycf3* genes containing two introns, the genes contained only one intron (Table 1). The *trnK-UUU* gene had the longest intron, which was 2578 bp. The *rps12* gene had a trans-spliced structure, with the 5′ end and 3′ end located in the LSC region and IR region, respectively, and the *rpl22* gene had similar structural characteristics.

**Table 1:** Introns and exons in genes in the chloroplast genome of “Shanxinyang”

Gene name	Exon	Intron	Exon	Intron	Exon
*ndhB*	755	681	776	–	–
*clpP*^a^	227	630	291	797	70
*rpl2*	433	668	390	–	–
*ycf3*^a^	154	725	225	732	125
*rpoC1*	1618	790	429	–	–
*atpF*	410	725	143	–	–
*ndhA*	546	1072	550	–	–
*rps12*	26	535	231	–	–
*trnA-UGC*	35	801	36	–	–
*trnE-UUC*	39	947	31	–	–
*trnK-UUU*	35	2578	37	–	–
*trnT-CGU*	34	693	42	–	–
*trnL-UAA*	34	599	49	–	–
*trnC-ACA*	55	581	38	–	–
*trnI-GAU*	35	15	35	–	–
*trnA-UGC*	36	39	27	–	–

Note: ^a^ indicates that there are 2 introns in this gene

### Repeat sequence analysis

According to the characteristics of the genome repeats, they could be divided into two categories: tandem repeats and interspersed repeats. The second category mainly consists of transposable elements (TEs). The first type of tandem repeat consists of microsatellites or simple sequence repeats (1–9 bases composing a repeat unit) and minisatellites (10–60 bases composing a repeat unit). In this study, 72 SSRs were detected in the “Shanxinyang” chloroplast genome, including 8 mononucleotide SSRs, 4 dinucleotide SSRs, 8 trinucleotide SSRs, 8 tetranucleotide SSRs, 13 pentanucleotide SSRs, 22 hexanucleotide SSRs, 7 heptanucleotide SSRs, 1 octonucleotide SSR and 1 nonanucleotide SSR (Table 2). Most SSRs were located in non-coding regions, and only a few SSRs were located in CDSs. Twenty-one pairs were detected in the “Shanxinyang” chloroplast genome (Table 2).

**Table 2:** Repeat sequence of the chloroplast genome of “Shanxinyang”

Repeat type	Subtype	Category: start-stop
SSR	1	(A)n:63961–63,979,98,244-98,268,73,696-73,721,73,480–73,506;(T)n:92750–92,768,74,999-75,023,49,077-49,105,110,784–110,815
	2	(AT)n:149467–149,490,90,969-90,996,55,839–55,888;(TA)n:62786–62,864
	3	(AAT)n:74499–74,548;(ATA)n:102256–102,285;(ATT)n:140391–140,419;(CTT)n:1437–1467;(TAA)n:70134–70,169;(TAT)n:54868–54,916,36,241–36,351;(TTT)n:82352–82,389
	4	(AAAC)n:74209–74,231;(AATA)n:86940–86,976;(ATTT)n:156029–156,084,42,745–42,828;(TAAA)n:96056–96,078;(TATT)n:17554–17,592,104,656–104,727;(TTAA)n:108558–108,614
	5	(AATGG)n:1766–1807;(AATTC)n:55169–55,234;(AATTT)n:2510–2537;(CATAT)n:95486–95,524;(TAACT)n:86305–86,367;(TCCAT)n:137687–137,728;(TCTAA)n:94997–95,064;(TGATA)n:20401–20,454;(TTATT)n:91555–91,597;(TTTAT)n:56856–56,898,99,816–99,902;(TTTTA)n:110753–110,814;(TTTTG)n:90467–90,497
	6	(AATATA)n:72201–72,248;(AATCAA)n:31871–31,900;(ACTAAT)n:11422–11,466;(ATAATG)n:93151–93,186;(ATAGAA)n:1684–1723;(ATATAG)n:105068–105,122;(ATATTA)n:97585–97,658;(ATTAAG)n:36305–36,369;(ATTAAT)n:59132–59,174;(TAAGAG)n:27396–27,435;(TATTAG)n:128028–128,072;(TATTTT)n:92995–93,038;(TCTTTC)n:79848–79,887;(TGTTTC)n:813–877;(TTAATA)n:33386–33,431;(TTTATA)n:136950–136,956;(TTTATT)n:127492–127,541;(TTTCTA)n:137771–137,810;(TTTGTT)n:75579–75,618;(TTTTAT)n:84635–84,674;(TTTTGC)n:70652–70,685;(TTTTTG)n:78823–78,863
	7	(AATATTA)n:95525–95,537,95,464–95,485;(ATAATAA)n:93187–93,196;(ATTCTAA)n:63445–63,546;(TAATAGG)n:63677–63,711;(TTATTAT)n:27333–27,389;(TTATTTT)n:39270–39,354
	8	(TATTTGTA)n:109761–109,843
	9	(AATAAATAG)n:58568–58,608
Low_complexity	A-rich	< 30:2538–2557,11,957–12,005;30–80:27674–27,709,44,037-44,073,49,380-49,446,54,158-54,193,57,311-57,358,74,380-74,429,92,835-92,880,93,954-94,030,94,879-94,922,99,929-99,969,112,105-112,161,121,902-121,940,136,957–136,984; > 80:139622–139,711,146,459–146,569
	GA-rich	30–40:138027–138,057,139,634–139,678
LTR/Gypsy	Atlantys3_I-int	37: 24992–25,029,114,465–114,502
rRNA	LSU-rRNA_Ath	30–80:3674–3910; > 80:4349–4462,4612-4659,5526-5687,5829-5931,6090–6217
	SSU-rRNA_Ath	73:8893–8966,9062–9417; 134:9482–9616,129,878–130,012; 355:130077–130,432,130,528–130,601
	LSU-rRNA_Ath	47:133277–133,404; > 80:133563–133,665,133,807-133,968,134,835-134,882,135,032-135,145,135,584–135,820

Note: All the repeat positions are listed with the start and stop positions

The second type of TEs includes two types: retroelements of class-I TEs transposed by RNA-mediated mechanisms and DNA transposons of class-II TEs transposed by DNA. Class I TEs are mainly composed of LTRs (long terminal repeats). Some sequences of LTRs may have encoding functions. Two LTR/Gypsy elements were found in the chloroplast genome, and 18 rRNA-like repeats were found (Table 2).

### Chloroplast transcriptome analysis

**Fig. 2:**
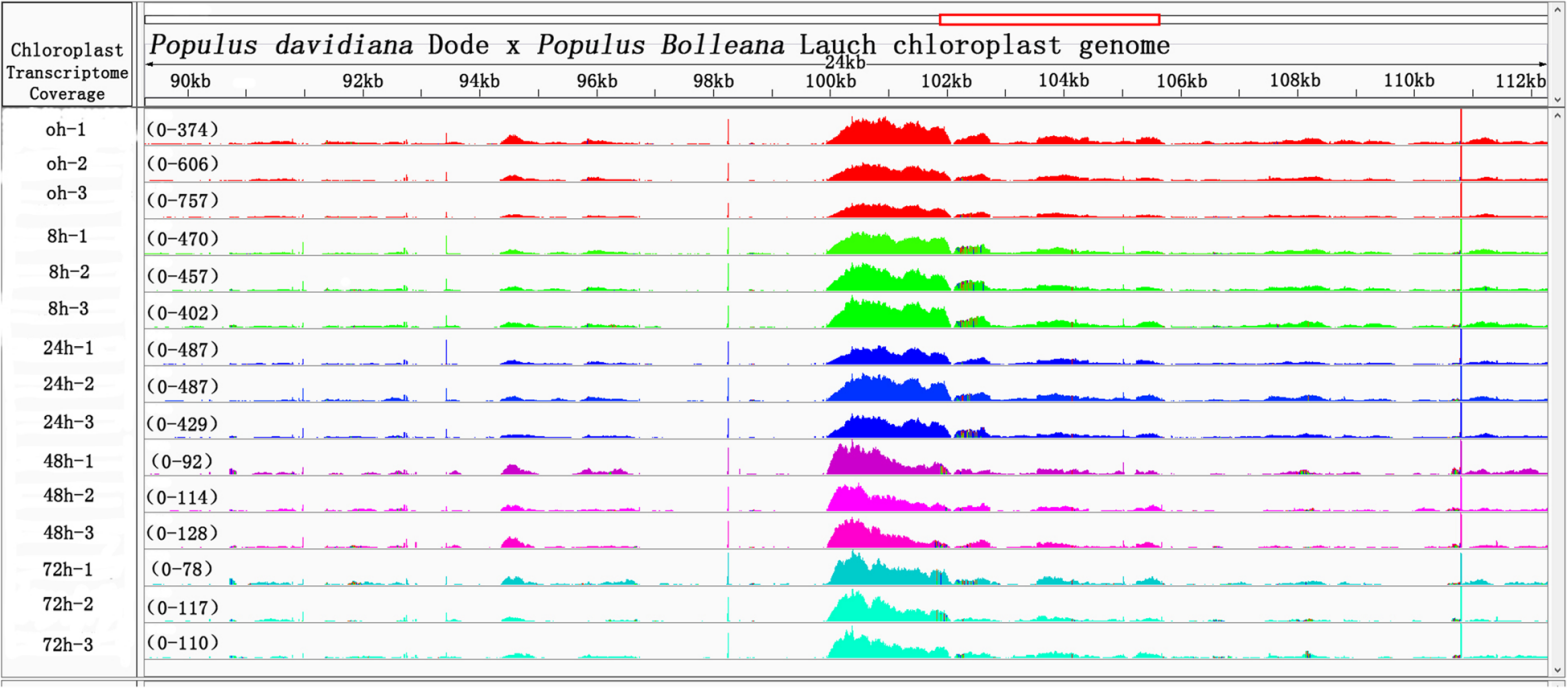
Comparison of the “Shanxinyang” transcriptome to the chloroplast

Sequence data were collected from the samples 0, 8, 24, 48 and 72 h after 20% PEG treatment and mapped to the chloroplast genome as shown in Fig. 2. Bowtie2 was used to compare the reads obtained from each sample with the chloroplast genome. Then, the read counts were used to calculate the expression level.

**Fig. 3:**
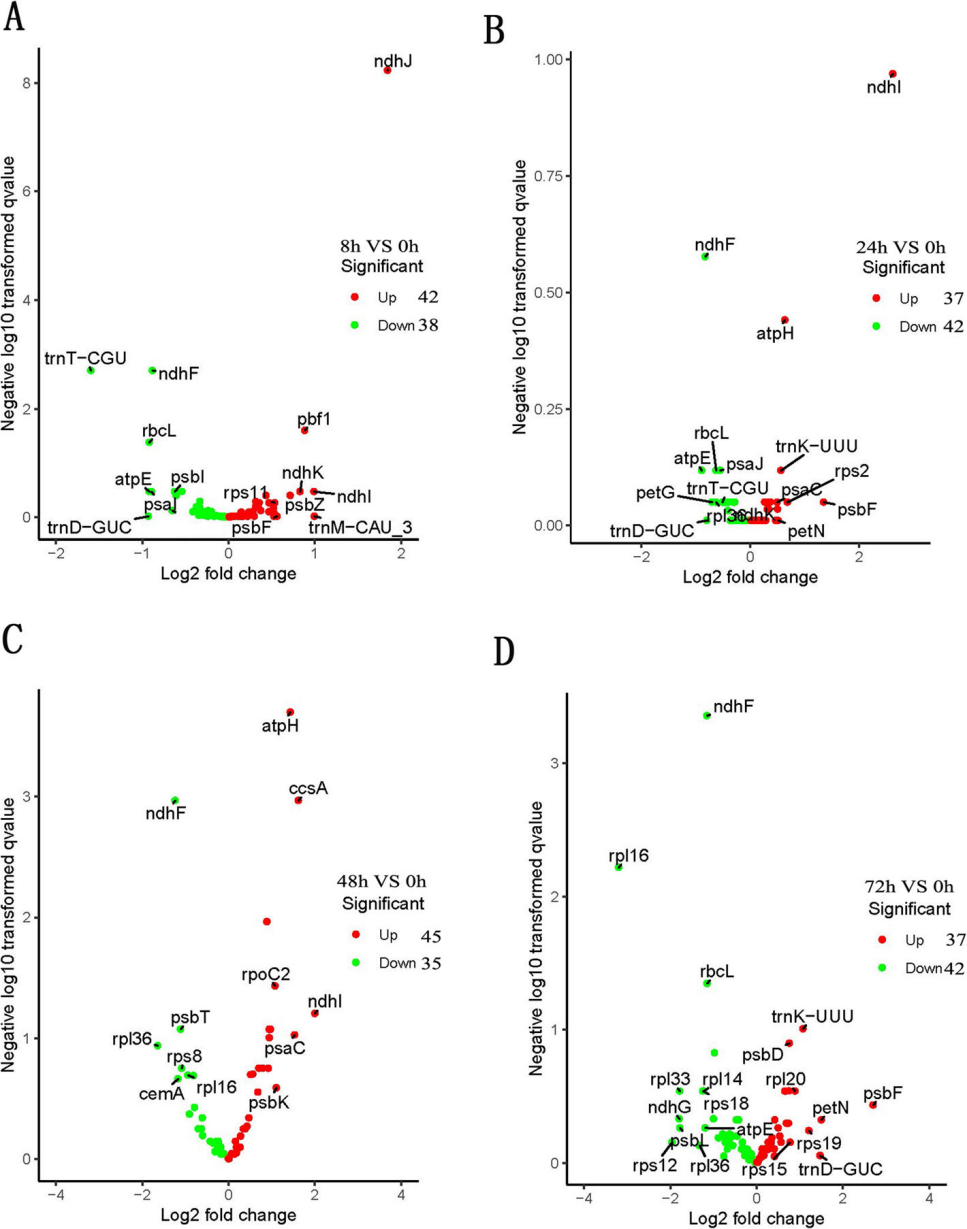
The cpDEGs in the cp transcriptome of “Shanxinyang”. All the treatment groups (8, 24, 48 and 72 h) was compared with the CK group (0 h); The cp DEGs of each group were shown by the volcano maps; **a**: 8 h VS 0 h; **b**: 24 h VS 0 h; **c**: 48 h VS 0 h; **d**: 72 h VS 0 h

According to the comparison results, the expression level was estimated. Log2 (fold change) was used to express the expression abundance of the transcripts. Volcano plots were used to examine differences between groups, and statistical significance was estimated by DESeq (fold change (> 2); FDR < 0.01). Volcano plots of the 4 groups are shown in Fig. 3a, b, c and d. In total, we found 42 up-regulated and 38 down-regulated genes in the 8 h VS 0 h group comparison (Fig. 3a); 37 up-regulated and 42 down-regulated genes in the 24 h VS 0 h group comparison (Fig. 3b); 45 up-regulated and 35 down-regulated genes in the 48 h VS 0 h group comparison (Fig. 3c); and 37 up-regulated and 42 down-regulated genes in the 72 h VS 0 h group comparison (Fig. 3d). Drought stress regulation of key genes was found in further analyses, in which the cpDEGs were involved in drought responses. Moreover, we sumarized the detail information of the up regulated and down regulated cp DEGs in Table S2, which may be functional to the drought defence. Further more, the lasting regulated cp DEGs was shown in the Venn maps (Figure S1). Among these genes, most were the key genes to form the photosynthetic systems, and some tRNAs were also found. In which, the *rpl16*, *petG*, *atpE*, *psaI*, *rps18*, *psbL*, *rps19*, *rpl14*, *trnL-UAA*, *rpl22*, *rbcL*, *trnT-CGU* and *psaB* were found lasting down regulated. While, the *psbF*, *petN*, *psbM*, *rpoC1*, *rps11*, *ndhC*, *rps19*, *ndhK*, *trnK-UUU*, *psbB*, *atpI* and *ycf3* were found up regulated.

**Fig. 4:**
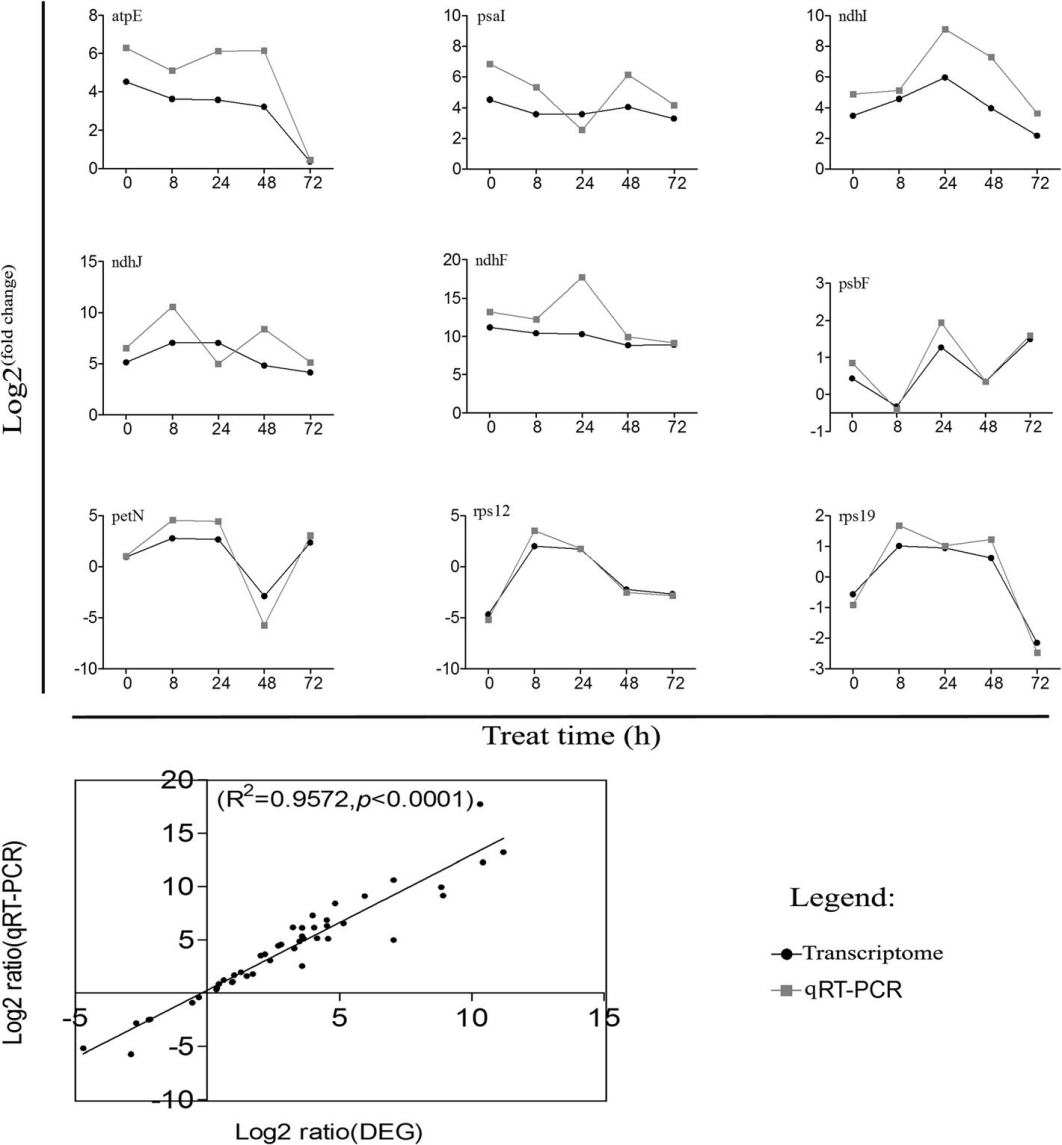
RT–qPCR of cpDEGs in the chloroplast transcriptome of “Shanxinyang”

To further validate this approach, we selected 9 cpDEGs to conduct reverse transcription (RT)–qPCR (Fig. 4), which indicated that the results were reliable because the Log2 (fold change) was significantly consistent with the qPCR results.

### Phylogenetic analysis

**Fig. 5:**
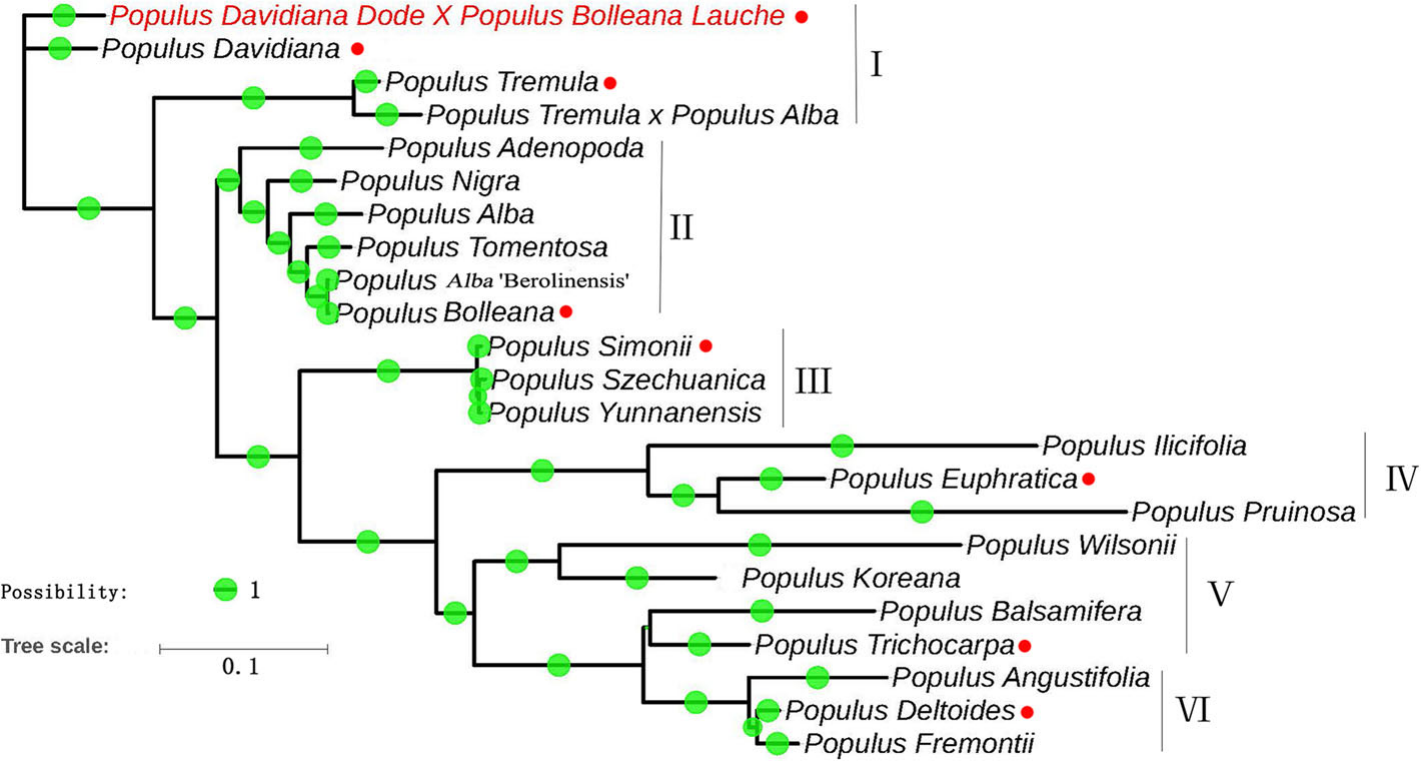
Phylogenetic tree of *Populus* plants. Red dot: 7 representative species from 4 sections

In this study, published chloroplast genomes of 23 *Populus* species were used for phylogenetic analysis. The phylogenetic position of “Shanxinyang” in *Populus* was determined by constructing a phylogenetic tree. The phylogenetic tree had 6 branches, labelled clades I - VI (Fig. 5). Clades I and II belonged to Sect. *Populus*. Clades III and V belonged to Sect. *Tacamahaca.* Clade IV belonged to Sect. *Turanga*, and clade VI belonged to Sect. *Aigeiros*. “Shanxinyang” and *Populus davidiana* were on sister branches that belonged to clade I. At the same time, we fould differences between “Shanxinyang” and *Populus* species in phylogenetic analysis, which guide us to think the sequences details of the chloroplasts IRs, non-coding and coding regions, which may explaine the unique systematic and evolutionary positions of “Shanxinyang” and partly explained its superior stress tolerance compared with that of other *Populus* plants and may guide insight to the further drought stress regulation of key cpDEGs.

### Analysis of IR region contraction and expansion

**Fig. 6:**
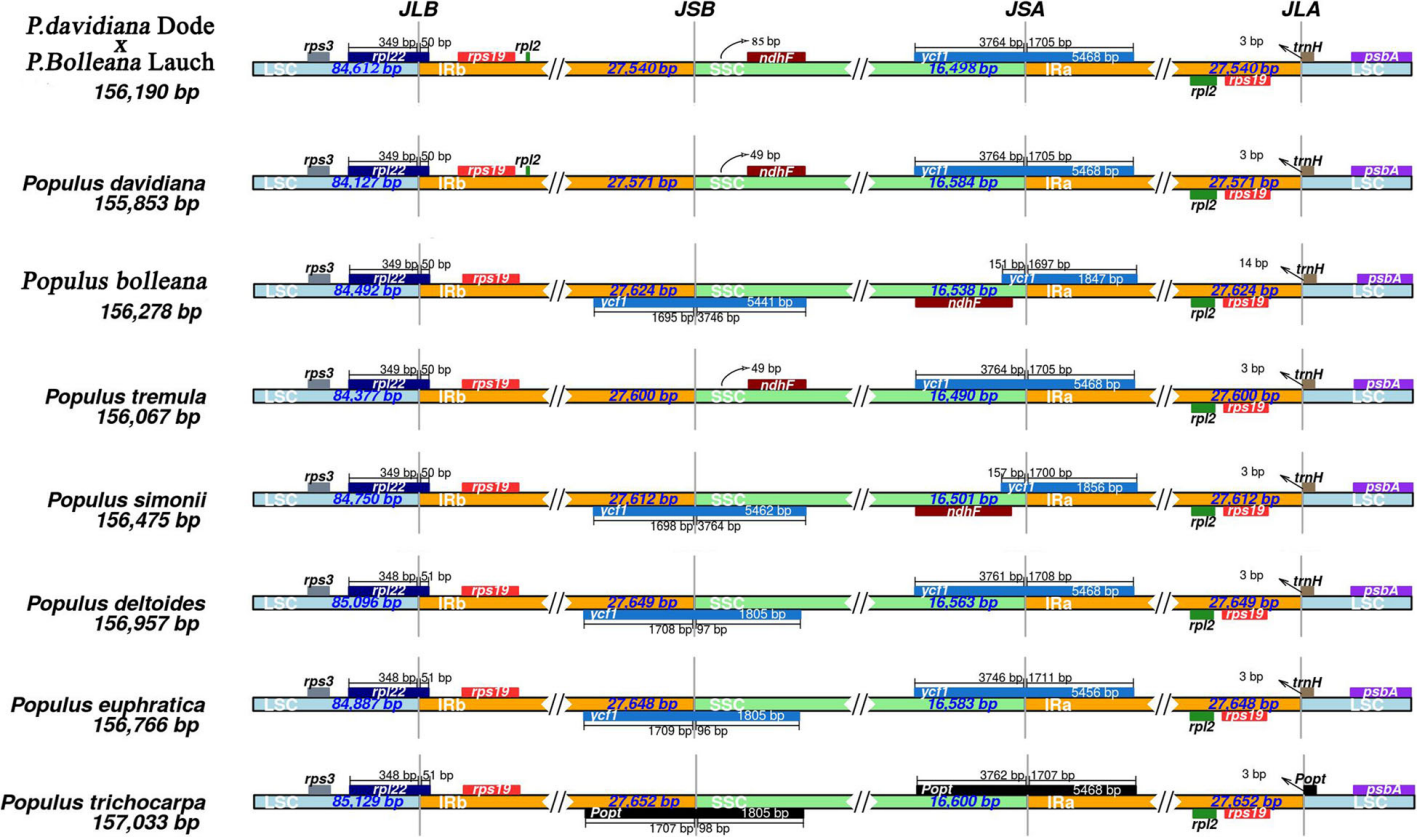
Analysis of IR regions of *Populus* chloroplast genomes

The contraction and expansion of the IR regions may be the main reasons for the diversity of chloroplast genome length in angiosperms (Kim and Lee, 2004). Comparing the IR regions of “Shanxinyang” and other poplar chloroplast genomes with the boundaries of the LSC and SSC regions, it was found that four junctions (JLA, JLB, JSA and JSB) were located between the two IRs (IRB and IRA) and the two single-copy regions (LSC and SSC), and the IR region of the chloroplast genome in *Populus* was found to have experienced contraction and expansion (Fig. 6). The LSC-IRb junction of all 8 plants was between the *rps19* and *rpl22* genes. The LSC-IRa junction was between the *rps19* and *trnH* genes because the IR region did not expand at the IRa-LSC junction, such that the *trnH* gene was still in the LSC region, but most of them were close to the boundary (< 10 bp). The IR region of *Populus* plants shrank obviously at the LSC-IRb junction, which led to the generation of a *ycf1* pseudogene in the IRa region of these species.

### Comparative analysis of *Populus* chloroplast genomes

To determine the differences between “Shanxinyang” and other *Populus* species, the chloroplast genome of “Shanxinyang” was compared with that of 7 representative species from 4 sections. The sequence and composition of the chloroplast genome of “Shanxinyang” were most similar to those of *P. davidiana*. The chloroplast genome lengths of the *Populus* species were significantly different, with an average length of 157,727 bp. *P. deltoides* had the longest chloroplast genome, at 156,957 bp, and *P. davidiana* had the shortest chloroplast genome, with a difference of 1101 bp (Table 3). “Shanxinyang” had the most similar chloroplast genome to *Populus davidiana*, with the largest difference from *P. deltoides*.

**Table 3:** Comparison of *Populus chloroplast* genomes

Species	Size (bp)				G + C (%)	Number of genes				
	Total	LSC	IR	SSC		Protein-coding genes	rRNAs	tRNAs	Coding ratio (%)	GenBank accessions
*P. davidiana* Dode× *P. bolleana* Lauch	156,190	84,612	27,540	16,498	36.75	86	8	37	50.20	MN190025
*P. bolleana*	156,278	84,492	27,624	16,538	37.00	86	8	37	51.78	MK267319
*P. davidiana*	155,853	84,127	27,571	16,584	36.98	88	8	37	50.17	KX306825
*P. tremula*	156,067	84,377	27,600	16,490	36.80	86	8	37	65.65	KP861984
*P. simonii*	156,475	84,750	27,612	16,501	36.99	86	8	37	46.96	MK267304
*P. deltoides*	156,957	85,096	27,649	16,563	36.87	86	8	37	51.55	MK267316
*P. euphratica*	156,766	84,888	27,642	16,593	36.40	84	8	36	65.63	KJ624919
*P. trichocarpa*	157,033	85,129	27,652	16,600	36.70	100	8	37	68.97	EF489041

**Fig. 7:**
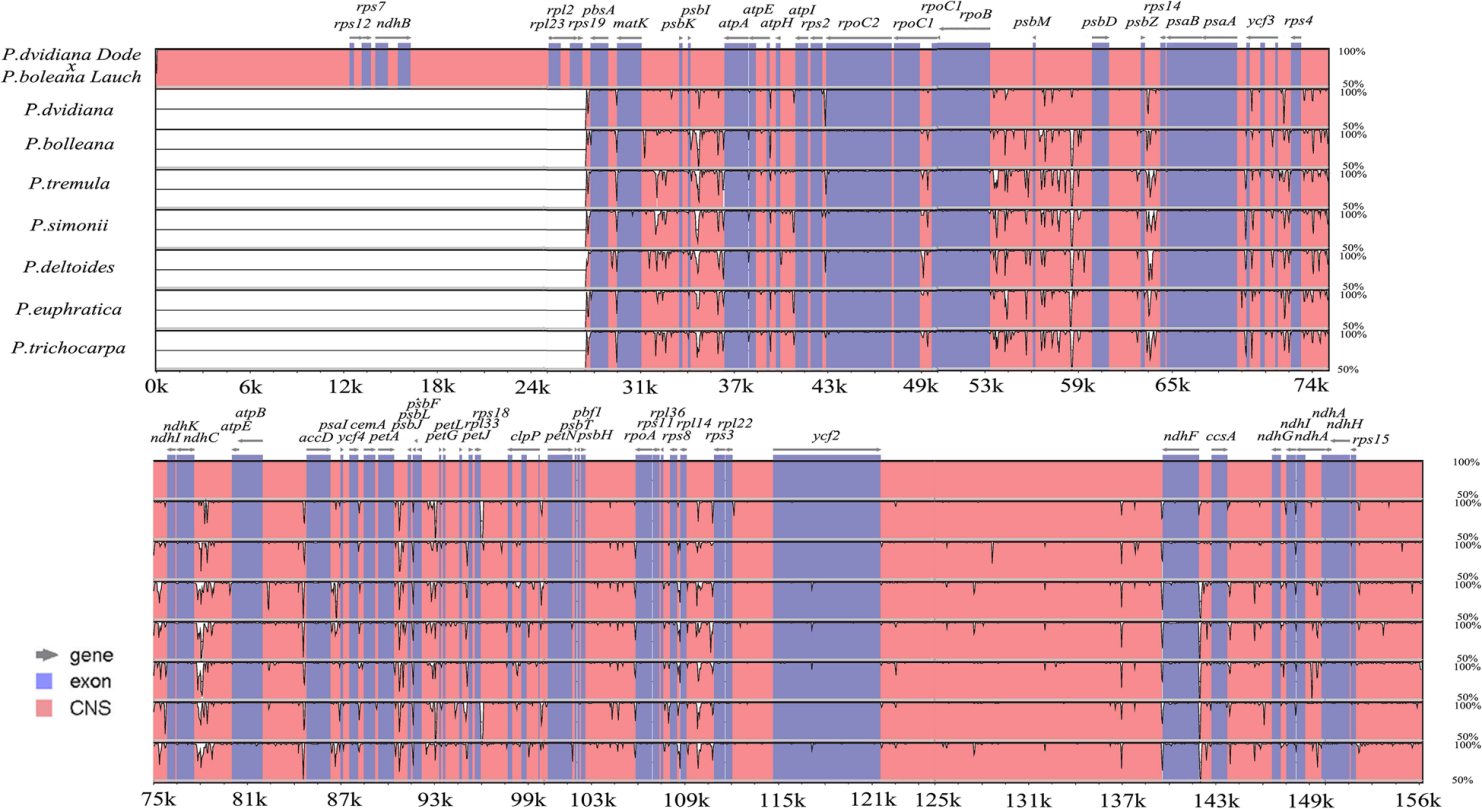
Sequence alignment of 8 related chloroplast genomes using the mVISTA program with “Shanxinyang” as a reference. Grey arrows above the alignment indicate the transcriptional directions of genes. Genome regions are colour coded as exons and conserved non-coding sequences (CNSs). A 50% identity cut-off was used for the plots. The *Y*-axis indicates the percent identity between 50 and 100%

To study the similarities of and differences in cp genome sequences between “Shanxinyang” and related species, a comparison program was used to compare these sequences. “Shanxinyang” was used as the reference genome in the mVISTA tool (Fig. 7). In general, the cp genome sizes of these related materials were not significantly different, but partially incomparable regions were also found, which indicated the unique phylogenetic position of “Shanxinyang”. The overall pattern of similarity among these sequences was very high, with homology greater than 90%. As shown in Fig. 7, the structure of these cp genomes was conserved, with neither translocation nor inversion in the sequence. As expected, the coding regions were more conserved than the non-coding regions. More specifically, most of the highly polymorphic regions were located in the intergenic regions (such as *matK- psbI/K*-*atpA, rpoB*-*psbM*-*psbD*, *ycf2*-*ndhF* and *ndhC*-*atpE*), but *ycf* genes had higher variability. These regions may be undergoing more rapid nucleotide substitution at the species level, which indicates the potential regulatory differences in the non-coding regions of the *Populus* chloroplast genome.

## Discussion

With the development of sequencing technology, research on the chloroplast genome has entered a new stage. An in-depth understanding of the chloroplast genome will undoubtedly lead to a better understanding of plants. The chloroplast is an important organelle for photosynthesis and provides the material necessary for life activities. Due to its maternal genetic inheritance, the chloroplast genome can allow maternal characteristics to remain unchanged in offspring. The chloroplast genome is the second largest genome after the nuclear genome, and its structural complexity is far lower than that of the nuclear genome.

### Cp genome sequence analysis of *Populus davidiana* Dode×*Populus bolleana* Lauch

The chloroplast genome in “Shanxinyang” is similar to that in other *Populus* plants, which to some extent reflects the high conservation of the angiosperm chloroplast genome [23]. Interestingly, it has been reported that the *ycf15* gene exists in most angiosperms. In contrast, we found that the *ycf15* gene was absent in the “Shanxinyang” and *Populus davidiana* chloroplast genomes, which showed that the *ycf15* gene can be transcribed as one of the precursors of polycistronic transcripts, but its function is still unclear [24]. Loss of protein-coding genes in chloroplast genomes is common in angiosperms [25]. In the *Myrsinaceae* s. str. Clade, Yan et al. (2019) [26] reported that the *ycf15* gene was absent from five taxa (*Elingamita johnsonii, Tapeinosperma netor, Tapeinosperma multiflorum, Parathesis donnell-smithii* and *Parathesis chiapensis*).

SSRs have been widely used as gene markers in DNA fingerprinting, phylogenetics, species identification and population genetic diversity analysis due to their co-dominant inheritance and high polymorphism [27–29]. Herein, a total of 72 SSRs were identified in the “Shanxinyang” chloroplast genome, most of which were located in non-coding regions and a few of which were located in CDSs. A similar pattern was also observed in the *Loropetalum subcordatum* [30] and *Quercus bawanglingensis* chloroplast genomes [31].

In most angiosperms, the A/T content of SSRs is higher than the G/C content [32, 33]. A similar pattern was observed in “Shanxinyang”, with 8 single-A/T SSRs. In contrast to other plants, “Shanxinyang” has a higher A/T content. In this study, two LTR/Gypsy and 18 rRNA-like repeats were found in the “Shanxinyang” chloroplast genome. The long repeat sequence had a length greater than or equal to 30 bp and may function to promote the rearrangement of the chloroplast genome and increase genetic diversity [34].

### Phylogenetic tree based on *Populus* cp genomes

The chloroplast genome is of great value for understanding plant evolution [35]. In this investigation, “Shanxinyang” and *Populus davidiana* were located on sister branches, belonging to clade I (Sect. *Populus*), which was similar to the pattern in the APG IV classification system [11]. In addition, some studies have suggested that *Populus simonii* is located on a sister branch of *Populus trichocarpa* [9]; however, the results of this study showed that *Populus trichocarpa* was on a new branch (clade V), which was similar to the pattern in the APG IV classification system [11, 36]. The angiosperm *Populus trichocarpa* has long been considered to have originated in North America. In recent years, most studies have shown that *Populus trichocarpa* and *Populus simonii* are sister to each other, which together constitute Sect. *Tacamahaca*. The results of this study support “Shanxinyang” as belonging to Sect. *Populus* (clade I).

### IR region variation

The chloroplast genome has a high gene conversion ability, which ensures the consistency and stability of the two IR reverse-replication regions, thereby enhancing its own stability and conservation. Therefore, contractions and expansions of IR regions represent important evolutionary events that are responsible for the size variation in the cpDNA genome [37, 38]. During the continuous evolution of species, a large degree of variation remains in the IR regions of some species [39, 40]. In this study, the IR regions had a higher GC content than the other regions due to the presence of eight rRNA sequences in the IR regions [41, 42], and the lengths of the IR regions in *Populus* chloroplast genomes were generally similar. Therefore, IR region length is an important factor affecting the length of the chloroplast genome and is relatively conserved, which may be due to the copy correction caused by gene transformation between IR region sequences [39].

The IR regions are known to maintain the stability of the other regions in the chloroplast genome because of the intramolecular recombination between IR copies, therefore limiting recombination between the two single-copy regions [43]. Compared with that in other *Populus* chloroplast genomes, the IR region in the “Shanxinyang” chloroplast genome has expanded obviously, and *rpl22* and other genes are close to the LSC region. A similar pattern also occurs in the chloroplast genomes of other plants [44]. IR region expansion has been reported in many studies, and its mechanism has been discussed. It is believed that slight IR expansion may be caused by gene transfer, consistent with the pseudogenes found in the “Shanxinyang” and *P. davidiana* IR regions in this study, and greater IR expansion may be achieved through a double-strand break repair (DSBR) mechanism [45, 46]. However, there are few reports on IR region contraction, and at present, it is mainly believed that the IR region contraction mechanism is also a DSBR mechanism [44]. One of the reasons for a change in IR region length is recombination between repeat sequences or poly structures and tRNA [47].

### The Cp transcriptome and Populus cp genome comparison

Volcano plots of the 4 groups showed that approximately 35–50 cp genes were up-regulated or down-regulated in each group. Including 13 lasting down regulated and 12 up regulated genes. Among which, most were the key genes to form the photosynthetic systems, which partly explain the loss of green when the plants was drought, and some tRNAs were also found, which was important to the whole chloroplast biology. Some of these related proteins were also found to regulate plant defence [13–16]. These cp DEGs may be the potential ones to understand the chloroplast evolutionary biology to the drought in the further study. To further understand potential regulatory differences, plant systematic and evolutionary analyses should be performed with the sequences showing the greatest differences. According to mVISTA analysis, the structure of these cp genomes is conserved, and there is neither translocation nor inversion in the sequence. As expected, the coding regions are more conserved than the non-coding regions. More specifically, most of the highly polymorphic regions are located in intergenic regions. These regions may be undergoing more rapid nucleotide substitution at the species level, which indicates potential regulatory differences in the non-coding regions of the *Populus* chloroplast, explains the unique systematic and evolutionary positions of “Shanxinyang” and partly explains its superior stress tolerance compared with that of other *Populus* taxa. Drought stress regulation of key cpDEGs should be explored in further research.

## Conclusion

In this study, we completed the sequencing, assembly and annotation of the “Shanxinyang” chloroplast genome and analysed its structure, GC content, gene structure and repeat sequences. The results showed that the genome structure and gene composition of the “Shanxinyang” chloroplast were similar to those of most angiosperm chloroplasts, but the *ycf15* gene was missing. Compared with that in most *Populus* chloroplast genomes, the IR region in the “Shanxinyang” chloroplast genome was contracted. Through analysis of chloroplast genes that were differentially expressed under drought conditions, the expression mode of chloroplast genes under drought conditions was summarized. Through phylogenetic analysis of the chloroplast genome, the phylogenetic position of “Shanxinyang” was confirmed. Differences in chloroplast non-coding regions explained the unique systematic and evolutionary positions of “Shanxinyang” and partly explained its superior stress tolerance compared with that of other *Populus* plants and may guide further drought stress regulation of key cpDEGs. As a 2G plant, “Shanxinyang” has important scientific and economic value. The sequencing and analysis of its chloroplast genome also provide data for phylogenetic studies, species identification and stress resistance research in *Populus*.

## Methods

### Plant growth and material preparation

The *Populus davidiana Dode×Populus bolleana Lauch* materials used in the study were preserved in our laboratory and planted on the campus of Northeast Forestry University (latitude: 45°39′48.58″N, longitude: 126°36′59.77″E). As researchers in the laboratory, we performed hybridization and formal identification of the plant material used in this study, and we were allowed to use these materials for research. Sampling of these materials was performed in compliance with institutional, national, and international guidelines.

*Populus davidiana* Dode*×Populus bolleana* Lauch clones were surface sterilized in 3% H_2_O_2_, rinsed several times with sterile water, and germinated on rooting medium (4.4 g Murashige and Skoog medium, 3% sucrose, 0.7% agar, pH 5.8, 0.5 ppm NAA) for 4 weeks. Four-week-old plants were explanted on the rooting medium for another 4 weeks in a controlled environment (22–23 °C, 16 h light and 8 h dark). At this stage, the lines had grown to the same degree. Four-week-old plants were used for DNA isolation and stress treatment. Drought stress was induced by treatment with 20% polyethylene glycol (PEG 6000) as described by Kwon et al. (2014) [48]. The samples were collected 0, 8, 24, 48 and 72 h after 20% PEG 6000 treatments. Each group included 3 plantlets, which were quickly frozen in liquid nitrogen for DNA/RNA extraction and sequencing.

### DNA/RNA preparation, cDNA synthesis, library preparation and sequencing

DNA/RNA was isolated by plant kits (Qiagen, http,//www.qiagen.com). The total DNA/RNA was quantified by an Agilent 2100 Bioanalyzer (Agilent, http,//www.agilent.com). A DNA library was constructed, and read quality was evaluated according to Sharon et al. 2013 and Tilgner et al. 2014 [49, 50]. Further, RNA libraries were generated using a TruSeq RNA Library Prep Kit v2 (Illumina, USA), and cDNA was synthesized through freshly isolated mRNA. A HiSeq 2500 sequencer (Illumina, USA) was used for RNA-seq. At the same time, the Illumina RNA-seq data from 15 samples were evaluated by NGS QC Toolkit [51].

### Chloroplast genome assembly and annotation

With Illumina data, the genome was assembled using Velvet (version 1.2.10) [52] and NOVOPlasty (version 2.7.2) [53] software. Combining the two results, the optimal contig assembly was obtained. Then, SSPACE (version 3.0) [54] software was used to align the reads from all libraries to the contig sequence, and the scaffold sequence was further assembled by paired-end reads. Finally, GapFiller (1000 Genomes Project Consortium, 2012) (version 1–10) [55] software was used to align the reads of all libraries to the scaffold sequence, and the reads in the alignment were used to complement the gaps in the scaffold sequence. The scaffold sequence was extended. Finally, a scaffold sequence with a lower proportion of unknown (N) bases and a greater sequence length was obtained.

The chloroplast genome was annotated by GeSeq, an online tool. By BLAT with the reference genome, protein-coding genes, rRNA genes and tRNA genes were searched, and tRNA genes were annotated by tRNAscan-SE (v2.0) [56] and ARAGORN (v1.2) [57]. The online tool OGDRAW v1.2 (https://chlorobox.mpimp-golm.mpg.de/OGDraw.html) was used to draw a genome Circos map. For PCR validation, we designed PCR primers that covered boundary regions. The primers for each element are listed in Supplementary Table S1. PCR fragments were then sequenced.

RepeatModeler (version 1.0.8) software was used to predict repetitive sequences in the genome from scratch. The analysis was divided into two steps. The first step was to identify possible repetitive sequences by calling RECON (version 1.08) software and RepeatScout (version 1.0.5) software and then optimizing and constructing the results of the first step by using RepeatModeler software. The second step was to use RepeatMasker (version 4.0.5) software to search for and analyse repetitive sequences in the target genome. At the same time, NUCmer (NUCleotide MUMmer; version 3.1) was used to further detect the internal repeat sequence of the genome, and long repeat regions with lengths greater than or equal to 100 bp were screened.

### Chloroplast transcriptome and cpDEG qRT–PCR validation

The filtered RNA-seq reads were mapped to the corresponding plastome using Bowtie2. The following stringent alignment parameters were applied to properly align reads to the chloroplast genome: 1) reads that aligned to multiple genomic locations were ignored, and 2) for the uniquely mapped reads, tolerances were set to allow at most one mismatch. Then, the SAMtools package (version 1.9) was employed to index the alignment results as BAM files. The coverage and base depth were calculated by converting the BAM alignments into pileup files, which were then used for further statistical analyses of plastome transcription and visualisation in IGV 2.4.9.

Based on the plastome annotation files, we calculated the transcription of the different plastome genes. Position information for all coding regions (protein-coding, rRNA, and tRNA genes) and non-coding regions (intergenic regions and introns) was extracted from the annotation file. The transcription level for every genomic base-pair position was assigned on the basis of the number of sequence reads covering each position by HT-SEQ (version 0.11.2). Gene expression levels were estimated by DESeq2 (version 3.9) based on the reads from HT-SEQ [58]. The log2 of the score for each base-pair position of all the genes was calculated (Table S2), and volcano plots were created with R/Bioconductor.

Further, to verify the statistical significance and accuracy of the expression levels, cpDEGs were selected to conduct reverse-transcription PCR ((q)RT–PCR). First-strand cDNA was synthesized by a reverse transcription kit (TaKaRa). qRT-PCR primers were designed with SnapGene 2.0 (https,//www.snapgene.com/) (Table S1). qRT-PCR analysis was conducted in triplicate with *TdACTIN* (GenBank number MK273079) as a reference gene [59] by the qTOWER 2.0 platform (Analytic Jena, Germany). The reaction system for real-time PCR contained 10 μL of SYBR Green Real-time PCR Master Mix (TaKaRa), 0.5 μmol/L each forward and reverse primer, and 1 μL of template for a total volume of 20 μL. The PCR and 2^-ΔΔCt^ expression level calculations were performed according to Livak and Schmittgen (2001) [60].

### Polymorphism in the Populus chloroplast genome

Based on the APG IV system and the reported *Populus* chloroplast genomes, 23 complete chloroplast genomes of angiosperms were selected. A phylogenetic tree was constructed to identify the phylogenetic position of *Populus davidiana* Dode x *Populus bolleana* Lauche. Sequences were compared with ClustalW software. Phylogenetic trees were constructed by using MrBayes 3.2.2 of the XSEDE tools from the CIPRES Science Gateway server (http://www.phylo.org/). Phylogenetic trees were viewed by iTol2 (http://itol2.embl.de/upload.cgi). The similarity between chloroplast boundaries was analysed by IRscope (https://irscope.shinyapps.io/irapp/). The differences between chloroplast genomes were analysed by mVISTA (http://genome.lbl.gov/vista/mvista/submit.shtml).

### Statistical analysis

Data analysis was performed using SPSS version 11.5 (SPSS Inc., Chicago, IL, USA). Correlation analysis was performed to test for correlations between qRT-PCR and RNA-Seq data, the log2-fold change values of transcripts were plotted in GraphPad Prism 5, and corresponding R^2^ and *p* values were calculated. All plots were created using R packages and GraphPad Prism 5.

## Supplementary information

**Additional file 1 Table S1.** The sequences of primers used in boundary PCR and cpDEG qRT-PCR.

**Additional file 2 Table S2.** The information of cp DEGs and the expression levels.

**Additional file 3 Figure S1.** The Venn maps of the cp DEGs. A: up regulated genes; B: down regulated genes.

## Data Availability

Illumina HiSeq 2500 data have been submitted to the Sequence Read Archive (SRA) of the National Center for Biotechnology Information (NCBI) under accession number PRJNA534167. The complete cp genome of *Populus davidiana Dode×Populus bolleana Lauch* was submitted to GenBank under accession number MN190025.

## Abbreviations

APG: Angiosperm phylogeny group
CNS: Conserved non-coding sequence
cp: Chloroplast
IR: Inverted repeat
LSC: Large single copy
ML: Maximum likelihood
NCBI: National center for biotechnology information
SSC: Small single copy
SSR: Simple sequence repeat
