# Supplementary material for: Chloroplast (Cp) Transcriptome of P. davidiana Dode×P. bolleana Lauch provides insight into the Cp drought response and Populus Cp phylogeny
Source: BMC Evol Biol. 2020 May 6;20:51. doi: 10.1186/s12862-020-01622-7 (PMC7201580; doi:10.1186/s12862-020-01622-7)
Supplement: Supplementary file 1 — Additional file 1 Table S1. The sequences of primers used in boundary PCR and cpDEG qRT-PCR. [file 12862_2020_1622_MOESM1_ESM.docx]

**Table S1.** The sequences of primers used in boundary PCR and DEGs qRT-PCR.

| **Primer** | **Primer sequence (5'to3')** | **Illustration** |  | **Primer** | **Primer sequence (5'to3')** | **Illustration** |
| --- | --- | --- | --- | --- | --- | --- |
| LSC-IRb-F | CTTGAATCACATCGATTCG | PCR boundary between LSC and IRb |  | ndhJ-qF | CATACTTCTTCAGGTTGATC | qRT-PCR of ndhJ |
| LSC-IRb-R | CATATGAAGCATCCGCTTCAG | PCR boundary between LSC and IRb |  | ndhJ-qR | CCTCTGTCTGCTTGGCTGGTC | qRT-PCR of ndhJ |
| IRb-SSC-F | GACTAGATTGTGTAATGATG | PCR boundary between IRb and SSC |  | ndhF-qF | GCTTATATAAGGAAGACGC | qRT-PCR of ndhF |
| IRb-SSC-R | GATTGGTCCTATAATCGTGG | PCR boundary between IRb and SSC |  | ndhR-qR | ATTGGAGCTATAGGAATACC | qRT-PCR of ndhR |
| SSC-IRa-F | GGTAATTCATAAATAGATGAGTC | PCR boundary between SSC and IRa |  | psbF-qF | TTATCGTTGGATGAACTGC | qRT-PCR of psbF |
| SSC-IRa-R | CCGAGGATCAATGTACAG | PCR boundary between SSC and IRa |  | psbF-qR | ATGACTATAGATCGAACCTATC | qRT-PCR of psbF |
| IRa-LSC-F | CGTCGCCGCAGTAAATAGG | PCR boundary between IRa and LSC |  | petN-qF | ATGGATATAGTAAGTCTCGC | qRT-PCR of petN |
| IRa-LSC-R | GGCAGTGGATTGTGAATCC | PCR boundary between IRa and LSC |  | petN-qR | CTAGAGTCCACTTCTTCC | qRT-PCR of petN |
| atpE-qF | CTAGCTCGTCTGAGAGCTAG | qRT-PCR of atpE |  | rps12-qF | ATACACCCTAGTACATGTC | qRT-PCR of rps12 |
| atpE-qR | GGTATATTACCTAATCACG | qRT-PCR of atpE |  | rps12-qR | ATGCCAACTATTAAACAAC | qRT-PCR of rps12 |
| psaI-qF | ATGACAATTCTCAACAACTTAC | qRT-PCR of psaI |  | rps19-qF | AATAGTAACGTGGTCCCG | qRT-PCR of rps19 |
| psaI-qR | TTGAACATGAAGAGATAAAG | qRT-PCR of psaI |  | rps19-qR | CGGAAATTGAGAGTAGGTGC | qRT-PCR of rps19 |
| ndhI-qF | CTATAGGACATACACGAAC | qRT-PCR of ndhI |  | Actin-PF | CAACTGTTTCTTCAGAGAACC | Internal reference gene of qRT-PCR |
| ndhI-qR | GGTTCATGAATTATGGTCAAC | qRT-PCR of ndhI |  | Actin-PR | GGCTAGTATGGCACCTCCC | Internal reference gene of qRT-PCR |
|  |  |  |  |  |  |  |
