# Supplementary material for: Chloroplast (Cp) Transcriptome of P. davidiana Dode×P. bolleana Lauch provides insight into the Cp drought response and Populus Cp phylogeny
Source: BMC Evol Biol. 2020 May 6;20:51. doi: 10.1186/s12862-020-01622-7 (PMC7201580; doi:10.1186/s12862-020-01622-7)
Supplement: Supplementary file 2 — Additional file 2 Table S2. The information of cp DEGs and the expression levels. [file 12862_2020_1622_MOESM2_ESM.doc]

**Table S2. The information of cp DEGs and the expression levels.**

| Groups | Gene ID | Products (Full gene names) | Log2FoldChange | Significant |
| --- | --- | --- | --- | --- |
| 8 VS 0 h | trnT-CGU | tRNA-Thr | -1.593 | Down |
|  | trnD-GUC | tRNA-Asp | -0.926 | Down |
|  | rbcL | ribulose-1,5-bisphosphate carboxylase/oxygenase large subunit | -0.917 | Down |
|  | atpE | ATP synthase CF1 epsilon subunit | -0.917 | Down |
|  | psaI | photosystem I subunit VIII | -0.891 | Down |
|  | ndhF | NADH dehydrogenase subunit 5 | -0.880 | Down |
|  | psbL | photosystem II protein L | -0.650 | Down |
|  | psbI | photosystem II protein I | -0.621 | Down |
|  | ycf4 | photosystem I assembly protein Ycf4 | -0.603 | Down |
|  | trnC-ACA | tRNA-Cys | -0.537 | Down |
|  | psbJ | photosystem II protein J | -0.409 | Down |
|  | psaJ | photosystem I subunit IX | -0.369 | Down |
|  | psbA | ATP synthase CF1 alpha subunit | -0.359 | Down |
|  | petG | cytochrome b6/f complex subunit V | -0.340 | Down |
|  | psaB | photosystem I P700 chlorophyll a apoprotein A2 | -0.334 | Down |
|  | atpF | ATP synthase CF0 B subunit | -0.329 | Down |
|  | petA | cytochrome f | -0.313 | Down |
|  | rps18 | ribosomal protein S18 | -0.311 | Down |
|  | ndhG | NADH dehydrogenase subunit 6 | -0.286 | Down |
|  | atpB | ATP synthase CF1 beta subunit | -0.256 | Down |
|  | rpoA | RNA polymerase alpha subunit | -0.249 | Down |
|  | atpA | ATP synthase CF1 alpha subunit | -0.246 | Down |
|  | rps15 | ribosomal protein S15 | -0.238 | Down |
|  | psbC | photosystem II 44 kDa protein | -0.231 | Down |
|  | rpl36 | ribosomal protein L36 | -0.208 | Down |
|  | petD | cytochrome b6/f complex subunit IV | -0.203 | Down |
|  | trnL-UAA | tRNA-Leu | -0.194 | Down |
|  | rpl22 | ribosomal protein L22-fragment | -0.162 | Down |
|  | clpP | ATP-dependent Clp protease proteolytic subunit | -0.138 | Down |
|  | accD | acetyl-CoA carboxylase beta subunit | -0.106 | Down |
|  | rpl16 | ribosomal protein L16 | -0.097 | Down |
|  | cemA | envelope membrane protein | -0.072 | Down |
|  | rps19 | ribosomal protein S19 | -0.057 | Down |
|  | rpoB | RNA polymerase beta subunit | -0.050 | Down |
|  | rpl14 | ribosomal protein L14 | -0.025 | Down |
|  | petB | cytochrome b6 | -0.022 | Down |
|  | psbE | photosystem II protein V | -0.020 | Down |
|  | ndhD | NADH dehydrogenase subunit 4 | -0.006 | Down |
|  | psbM | photosystem II protein M | 0.010 | Up |
|  | rpl22 | ribosomal protein L22 | 0.017 | Up |
|  | trnT-UGU | tRNA-Thr | 0.023 | Up |
|  | psbD | photosystem II protein D2 | 0.039 | Up |
|  | rpoC1 | RNA polymerase beta' subunit | 0.051 | Up |
|  | atpI | ATP synthase CF0 A subunit | 0.062 | Up |
|  | ccsA | cytochrome c biogenesis protein | 0.065 | Up |
|  | rps4 | ribosomal protein S4 | 0.066 | Up |
|  | rps3 | ribosomal protein S3 | 0.071 | Up |
|  | psaA | photosystem I P700 chlorophyll a apoprotein A1 | 0.073 | Up |
|  | rpl33 | ribosomal protein L33 | 0.114 | Up |
|  | petL | cytochrome b6/f complex subunit VI | 0.138 | Up |
|  | ndhH | NADH dehydrogenase subunit 7 | 0.145 | Up |
|  | rps8 | ribosomal protein S8 | 0.149 | Up |
|  | psbB | photosystem II CP47 chlorophyll apoprotein | 0.150 | Up |
|  | rps14 | ribosomal protein S14 | 0.171 | Up |
|  | rps2 | ribosomal protein S2 | 0.173 | Up |
|  | rpl20 | ribosomal protein L20 | 0.191 | Up |
|  | psbK | photosystem II protein K | 0.224 | Up |
|  | rpoC2 | RNA polymerase beta'' subunit | 0.238 | Up |
|  | ycf3 | photosystem I assembly protein Ycf3 | 0.239 | Up |
|  | trnK-UUU | tRNA-Lys | 0.284 | Up |
|  | rps12 | ribosomal protein S12 | 0.301 | Up |
|  | ycf1 | hypothetical protein RF1 | 0.316 | Up |
|  | ndhA | NADH dehydrogenase subunit 1 | 0.320 | Up |
|  | atpH | ATP synthase CF0 C subunit | 0.347 | Up |
|  | psbH | photosystem II protein H | 0.357 | Up |
|  | ndhC | NADH dehydrogenase subunit 3 | 0.376 | Up |
|  | psbT | photosystem II protein T | 0.437 | Up |
|  | ndhE | NADH-plastoquinone oxidoreductase subunit 4L | 0.470 | Up |
|  | infA | translation initiation factor 1 | 0.471 | Up |
|  | psaC | photosystem I subunit VII | 0.523 | Up |
|  | rps19 | ribosomal protein S19 | 0.528 | Up |
|  | petN | cytochrome b6/f complex subunit VIII | 0.533 | Up |
|  | rps11 | ribosomal protein S11 | 0.538 | Up |
|  | psbF | photosystem II protein VI | 0.565 | Up |
|  | psbZ | photosystem II protein Z | 0.717 | Up |
|  | ndhK | NADH dehydrogenase subunit K | 0.831 | Up |
|  | pbf1 | photosystem biogenesis factor 1 | 0.885 | Up |
|  | ndhI | NADH dehydrogenase subunit I | 0.992 | Up |
|  | trnM-CAU | tRNA-Met | 0.997 | Up |
|  | ndhJ | NADH dehydrogenase subunit J | 1.846 | Up |
| 24 VS 0 h | atpE | ATP synthase CF1 epsilon subunit | -0.890 | Down |
|  | ndhF | NADH dehydrogenase subunit 5 | -0.827 | Down |
|  | trnD-GUC | tRNA-Asp | -0.796 | Down |
|  | petG | cytochrome b6/f complex subunit V | -0.702 | Down |
|  | rpl36 | ribosomal protein L36 | -0.630 | Down |
|  | rbcL | ribulose-1,5-bisphosphate carboxylase/oxygenase large subunit | -0.628 | Down |
|  | psaJ | photosystem I subunit IX | -0.551 | Down |
|  | trnT-CGU | tRNA-Thr | -0.513 | Down |
|  | petA | cytochrome f | -0.493 | Down |
|  | rpl16 | ribosomal protein L16 | -0.475 | Down |
|  | rpl22 | ribosomal protein L22 | -0.448 | Down |
|  | rps18 | ribosomal protein S18 | -0.431 | Down |
|  | psbA | ATP synthase CF1 alpha subunit | -0.420 | Down |
|  | psaI | photosystem I subunit VIII | -0.406 | Down |
|  | psbI | photosystem II protein I | -0.402 | Down |
|  | cemA | envelope membrane protein | -0.392 | Down |
|  | rpl22 | ribosomal protein L22 | -0.364 | Down |
|  | psbL | photosystem II protein L | -0.330 | Down |
|  | psbC | photosystem II 44 kDa protein | -0.313 | Down |
|  | psbD | photosystem II protein D2 | -0.284 | Down |
|  | ndhJ | NADH dehydrogenase subunit J | -0.263 | Down |
|  | psbJ | photosystem II protein J | -0.232 | Down |
|  | rpoA | RNA polymerase alpha subunit | -0.204 | Down |
|  | trnL-UAA | tRNA-Leu | -0.160 | Down |
|  | ycf4 | photosystem I assembly protein Ycf4 | -0.153 | Down |
|  | rps12 | ribosomal protein S12 | -0.130 | Down |
|  | rpoB | RNA polymerase beta subunit | -0.120 | Down |
|  | psaA | photosystem I P700 chlorophyll a apoprotein A1 | -0.116 | Down |
|  | psaB | photosystem I P700 chlorophyll a apoprotein A2 | -0.102 | Down |
|  | rpoC2 | RNA polymerase beta'' subunit | -0.087 | Down |
|  | trnC-ACA | tRNA-Cys | -0.081 | Down |
|  | psbE | photosystem II protein V | -0.074 | Down |
|  | atpB | ATP synthase CF1 beta subunit | -0.061 | Down |
|  | petB | cytochrome b6 | -0.057 | Down |
|  | petL | cytochrome b6/f complex subunit VI | -0.052 | Down |
|  | clpP | ATP-dependent Clp protease proteolytic subunit | -0.040 | Down |
|  | rps19 | ribosomal protein S19 | -0.040 | Down |
|  | rps3 | ribosomal protein S3 | -0.038 | Down |
|  | accD | acetyl-CoA carboxylase beta subunit | -0.030 | Down |
|  | rpl33 | ribosomal protein L33 | -0.025 | Down |
|  | rpl20 | ribosomal protein L20 | -0.016 | Down |
|  | rpl14 | ribosomal protein L14 | -0.009 | Down |
|  | petD | cytochrome b6/f complex subunit IV | 0.013 | Up |
|  | rps8 | ribosomal protein S8 | 0.054 | Up |
|  | rpoC1 | RNA polymerase beta' subunit | 0.068 | Up |
|  | trnT-UGU | tRNA-Thr | 0.077 | Up |
|  | ndhC | NADH dehydrogenase subunit 3 | 0.085 | Up |
|  | ndhD | NADH dehydrogenase subunit 4 | 0.100 | Up |
|  | psbM | photosystem II protein M | 0.112 | Up |
|  | psbZ | photosystem II protein Z | 0.114 | Up |
|  | psbB | photosystem II CP47 chlorophyll apoprotein | 0.118 | Up |
|  | infA | translation initiation factor 1 | 0.122 | Up |
|  | atpF | ATP synthase CF0 B subunit | 0.127 | Up |
|  | psbK | photosystem II protein K | 0.155 | Up |
|  | psbH | photosystem II protein H | 0.170 | Up |
|  | ycf1 | hypothetical protein RF1 | 0.186 | Up |
|  | rps4 | ribosomal protein S4 | 0.195 | Up |
|  | ndhG | NADH dehydrogenase subunit 6 | 0.197 | Up |
|  | psbT | photosystem II protein T | 0.209 | Up |
|  | ccsA | cytochrome c biogenesis protein | 0.221 | Up |
|  | ndhH | NADH dehydrogenase subunit 7 | 0.257 | Up |
|  | ndhE | NADH-plastoquinone oxidoreductase subunit 4L | 0.264 | Up |
|  | rps14 | ribosomal protein S14 | 0.266 | Up |
|  | atpA | ATP synthase CF1 alpha subunit | 0.269 | Up |
|  | rps11 | ribosomal protein S11 | 0.287 | Up |
|  | pbf1 | photosystem biogenesis factor 1 | 0.314 | Up |
|  | ndhA | NADH dehydrogenase subunit 1 | 0.329 | Up |
|  | atpI | ATP synthase CF0 A subunit | 0.342 | Up |
|  | ycf3 | photosystem I assembly protein Ycf3 | 0.370 | Up |
|  | rps19 | ribosomal protein S19 | 0.470 | Up |
|  | rps15 | ribosomal protein S15 | 0.485 | Up |
|  | psaC | photosystem I subunit VII | 0.496 | Up |
|  | ndhK | NADH dehydrogenase subunit K | 0.503 | Up |
|  | petN | cytochrome b6/f complex subunit VIII | 0.513 | Up |
|  | trnK-UUU | tRNA-Lys | 0.565 | Up |
|  | atpH | ATP synthase CF0 C subunit | 0.637 | Up |
|  | rps2 | ribosomal protein S2 | 0.683 | Up |
|  | psbF | photosystem II protein VI | 1.349 | Up |
|  | ndhI | NADH dehydrogenase subunit I | 2.621 | Up |
| 48 VS 0 h | rps19 | ribosomal protein S19 | -2.826 | Down |
|  | rpl16 | ribosomal protein L16 | -2.094 | Down |
|  | ndhI | NADH dehydrogenase subunit I | -1.931 | Down |
|  | psaC | photosystem I subunit VII | -1.667 | Down |
|  | psbL | photosystem II protein L | -1.572 | Down |
|  | ndhG | NADH dehydrogenase subunit 6 | -1.457 | Down |
|  | ccsA | cytochrome c biogenesis protein | -1.251 | Down |
|  | rpl33 | ribosomal protein L33 | -1.091 | Down |
|  | atpE | ATP synthase CF1 epsilon subunit | -1.010 | Down |
|  | rpl14 | ribosomal protein L14 | -0.948 | Down |
|  | rbcL | ribulose-1,5-bisphosphate carboxylase/oxygenase large subunit | -0.902 | Down |
|  | trnF-GAA | tRNA-Phe | -0.870 | Down |
|  | trnT-UGU | tRNA-Thr | -0.844 | Down |
|  | rps18 | ribosomal protein S18 | -0.799 | Down |
|  | pbf1 | photosystem biogenesis factor 1 | -0.796 | Down |
|  | psaI | photosystem I subunit VIII | -0.750 | Down |
|  | psaJ | photosystem I subunit IX | -0.720 | Down |
|  | petG | cytochrome b6/f complex subunit V | -0.713 | Down |
|  | rpl22 | ribosomal protein L22 | -0.628 | Down |
|  | rpoC2 | RNA polymerase beta'' subunit | -0.624 | Down |
|  | rps3 | ribosomal protein S3 | -0.593 | Down |
|  | atpH | ATP synthase CF0 C subunit | -0.570 | Down |
|  | atpB | ATP synthase CF1 beta subunit | -0.566 | Down |
|  | ndhH | NADH dehydrogenase subunit 7 | -0.471 | Down |
|  | rps4 | ribosomal protein S4 | -0.457 | Down |
|  | psbK | photosystem II protein K | -0.413 | Down |
|  | rps14 | ribosomal protein S14t | -0.401 | Down |
|  | rpl22 | ribosomal protein L22-fragment | -0.371 | Down |
|  | ycf1 | hypothetical protein RF1 | -0.326 | Down |
|  | psbA | ATP synthase CF1 alpha subunit | -0.275 | Down |
|  | petL | cytochrome b6/f complex subunit VI | -0.254 | Down |
|  | clpP | ATP-dependent Clp protease proteolytic subunit | -0.242 | Down |
|  | trnC-ACA | tRNA-Cys | -0.210 | Down |
|  | psbE | photosystem II protein V | -0.183 | Down |
|  | ndhA | NADH dehydrogenase subunit 1 | -0.136 | Down |
|  | trnL-UAA | tRNA-Leu | -0.125 | Down |
|  | psbH | photosystem II protein H | -0.124 | Down |
|  | atpF | ATP synthase CF0 B subunit | -0.114 | Down |
|  | accD | acetyl-CoA carboxylase beta subunit | -0.092 | Down |
|  | trnT-CGU | tRNA-Thr | -0.090 | Down |
|  | rps2 | ribosomal protein S2 | -0.080 | Down |
|  | psaB | photosystem I P700 chlorophyll a apoprotein A2 | -0.052 | Down |
|  | psaA | photosystem I P700 chlorophyll a apoprotein A1 | -0.004 | Down |
|  | psbD | photosystem II protein D2 | -0.004 | Down |
|  | ndhE | NADH-plastoquinone oxidoreductase subunit 4L | 0.000 | Down |
|  | ndhD | NADH dehydrogenase subunit 4 | 0.008 | Up |
|  | psbB | photosystem II CP47 chlorophyll apoprotein | 0.084 | Up |
|  | rpl20 | ribosomal protein L20 | 0.095 | Up |
|  | ndhJ | NADH dehydrogenase subunit J | 0.117 | Up |
|  | rpoB | RNA polymerase beta subunit | 0.119 | Up |
|  | petD | cytochrome b6/f complex subunit IV | 0.124 | Up |
|  | rpoC1 | RNA polymerase beta' subunit | 0.156 | Up |
|  | psbC | photosystem II 44 kDa protein | 0.184 | Up |
|  | ndhF | NADH dehydrogenase subunit 5 | 0.203 | Up |
|  | psbJ | photosystem II protein J | 0.237 | Up |
|  | ndhK | NADH dehydrogenase subunit K | 0.245 | Up |
|  | trnK-UUU | tRNA-Lys | 0.260 | Up |
|  | petB | cytochrome b6 | 0.302 | Up |
|  | atpA | ATP synthase CF1 alpha subunit | 0.309 | Up |
|  | ycf4 | photosystem I assembly protein Ycf4 | 0.325 | Up |
|  | ycf3 | photosystem I assembly protein Ycf3 | 0.338 | Up |
|  | infA | translation initiation factor 1 | 0.411 | Up |
|  | rpl36 | ribosomal protein L36 | 0.427 | Up |
|  | psbI | photosystem II protein I | 0.472 | Up |
|  | cemA | envelope membrane protein | 0.490 | Up |
|  | psbM | photosystem II protein M | 0.533 | Up |
|  | petA | cytochrome f | 0.581 | Up |
|  | psbZ | photosystem II protein Z | 0.595 | Up |
|  | rps15 | ribosomal protein S15 | 0.737 | Up |
|  | rps8 | ribosomal protein S8 | 0.771 | Up |
|  | atpI | ATP synthase CF0 A subunit | 0.808 | Up |
|  | rps19 | ribosomal protein S19 | 0.905 | Up |
|  | rpoA | RNA polymerase alpha subunit | 0.999 | Up |
|  | psbT | photosystem II protein T | 1.028 | Up |
|  | rps11 | ribosomal protein S11 | 1.044 | Up |
|  | ndhC | NADH dehydrogenase subunit 3 | 1.592 | Up |
|  | psbF | photosystem II protein VI | 2.286 | Up |
|  | petN | cytochrome b6/f complex subunit VIII | 3.876 | Up |
| 72 VS 0 h | rpl16 | ribosomal protein L16 | -3.191 | Down |
|  | rps12 | ribosomal protein S12 | -1.955 | Down |
|  | ndhG | NADH dehydrogenase subunit 6 | -1.790 | Down |
|  | rpl33 | ribosomal protein L33 | -1.777 | Down |
|  | psbL | photosystem II protein L | -1.772 | Down |
|  | rpl36 | ribosomal protein L36 | -1.319 | Down |
|  | rps18 | ribosomal protein S18 | -1.241 | Down |
|  | rpl14 | ribosomal protein L14 | -1.221 | Down |
|  | atpE | ATP synthase CF1 epsilon subunit | -1.187 | Down |
|  | ndhF | NADH dehydrogenase subunit 5 | -1.147 | Down |
|  | rbcL | ribulose-1,5-bisphosphate carboxylase/oxygenase large subunit | -1.142 | Down |
|  | pbf1 | photosystem biogenesis factor 1 | -0.994 | Down |
|  | rps3 | ribosomal protein S3 | -0.970 | Down |
|  | ndhH | NADH dehydrogenase subunit 7 | -0.878 | Down |
|  | cemA | envelope membrane protein | -0.781 | Down |
|  | psbJ | photosystem II protein J | -0.773 | Down |
|  | rps19 | ribosomal protein S19 | -0.754 | Down |
|  | psaI | photosystem I subunit VIII | -0.717 | Down |
|  | psbZ | photosystem II protein Z | -0.691 | Down |
|  | ndhE | NADH-plastoquinone oxidoreductase subunit 4L | -0.671 | Down |
|  | rpl22 | ribosomal protein L22 | -0.640 | Down |
|  | trnT-CGU | tRNA-Thr | -0.636 | Down |
|  | rpl22 | ribosomal protein L22 | -0.617 | Down |
|  | ycf4 | photosystem I assembly protein Ycf4 | -0.603 | Down |
|  | rps4 | ribosomal protein S4 | -0.553 | Down |
|  | rps2 | ribosomal protein S2 | -0.547 | Down |
|  | atpF | ATP synthase CF0 B subunit | -0.462 | Down |
|  | rps8 | ribosomal protein S8 | -0.450 | Down |
|  | trnL-UAA | tRNA-Leu | -0.412 | Down |
|  | infA | translation initiation factor 1 | -0.343 | Down |
|  | rps14 | ribosomal protein S14 | -0.321 | Down |
|  | psaC | photosystem I subunit VII | -0.266 | Down |
|  | psbH | photosystem II protein H | -0.235 | Down |
|  | psbI | photosystem II protein I | -0.234 | Down |
|  | ndhA | NADH dehydrogenase subunit 1 | -0.227 | Down |
|  | psbT | photosystem II protein T | -0.209 | Down |
|  | petL | cytochrome b6/f complex subunit VI | -0.169 | Down |
|  | psbC | photosystem II 44 kDa protein | -0.115 | Down |
|  | psaB | photosystem I P700 chlorophyll a apoprotein A2 | -0.053 | Down |
|  | petB | cytochrome b6 | -0.040 | Down |
|  | ndhI | NADH dehydrogenase subunit I | -0.037 | Down |
|  | petG | cytochrome b6/f complex subunit V | -0.012 | Down |
|  | rps11 | ribosomal protein S11 | 0.016 | Up |
|  | trnC-ACA | tRNA-Cys | 0.029 | Up |
|  | psbE | photosystem II protein V | 0.036 | Up |
|  | rpoB | RNA polymerase beta subunit | 0.075 | Up |
|  | rpoA | RNA polymerase alpha subunit | 0.092 | Up |
|  | ycf1 | hypothetical protein RF1 | 0.126 | Up |
|  | psbB | photosystem II CP47 chlorophyll apoprotein | 0.135 | Up |
|  | psaJ | photosystem I subunit IX | 0.140 | Up |
|  | clpP | ATP-dependent Clp protease proteolytic subunit | 0.161 | Up |
|  | ndhJ | NADH dehydrogenase subunit J | 0.172 | Up |
|  | petA | cytochrome f | 0.211 | Up |
|  | atpA | ATP synthase CF1 alpha subunit | 0.213 | Up |
|  | ccsA | cytochrome c biogenesis protein | 0.259 | Up |
|  | psbA | ATP synthase CF1 alpha subunit | 0.274 | Up |
|  | atpB | ATP synthase CF1 beta subunit | 0.283 | Up |
|  | petD | cytochrome b6/f complex subunit IV | 0.283 | Up |
|  | rpoC2 | RNA polymerase beta'' subunit | 0.343 | Up |
|  | ndhD | cytochrome b6/f complex subunit VI | 0.361 | Up |
|  | ndhK | NADH dehydrogenase subunit K | 0.415 | Up |
|  | trnT-UGU | tRNA-Thr | 0.420 | Up |
|  | rpoC1 | RNA polymerase beta' subunit | 0.427 | Up |
|  | accD | acetyl-CoA carboxylase beta subunit | 0.504 | Up |
|  | atpI | ATP synthase CF0 A subunit | 0.541 | Up |
|  | psbK | photosystem II protein K | 0.574 | Up |
|  | ycf3 | photosystem I assembly protein Ycf3 | 0.659 | Up |
|  | psaA | photosystem I P700 chlorophyll a apoprotein A1 | 0.674 | Up |
|  | psbM | photosystem II protein M | 0.696 | Up |
|  | ndhC | NADH dehydrogenase subunit 3 | 0.744 | Up |
|  | atpH | ATP synthase CF0 C subunit | 0.750 | Up |
|  | psbD | photosystem II protein D2 | 0.764 | Up |
|  | rps15 | ribosomal protein S15 | 0.784 | Up |
|  | rpl20 | ribosomal protein L20 | 0.895 | Up |
|  | trnK-UUU | tRNA-Lys | 1.080 | Up |
|  | rps19 | ribosomal protein S19 | 1.218 | Up |
|  | trnD-GUC | tRNA-Asp | 1.477 | Up |
|  | petN | cytochrome b6/f complex subunit VIII | 1.504 | Up |
|  | psbF | photosystem II protein VI | 2.705 | Up |
