# Supplementary figures and images for: Chloroplast (Cp) Transcriptome of P. davidiana Dode×P. bolleana Lauch provides insight into the Cp drought response and Populus Cp phylogeny
Source: BMC Evol Biol. 2020 May 6;20:51. doi: 10.1186/s12862-020-01622-7 (PMC7201580; doi:10.1186/s12862-020-01622-7)

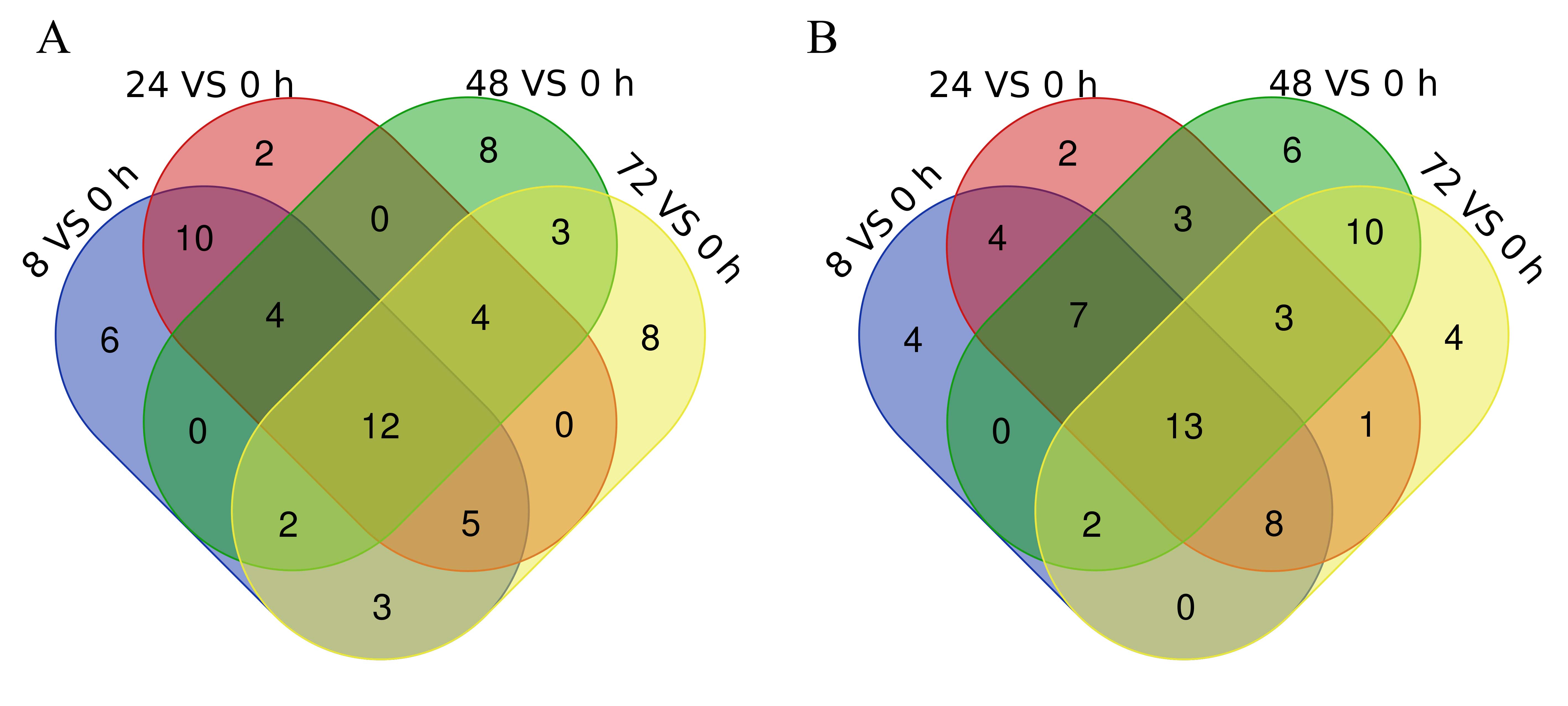

Supplement: Supplementary file 3 — Additional file 3 Figure S1. The Venn maps of the cp DEGs. A: up regulated genes; B: down regulated genes. [file 12862_2020_1622_MOESM3_ESM.jpg]
